# ACK1 is dispensable for development, skin tumor formation, and breast cancer cell proliferation

**DOI:** 10.1002/2211-5463.13149

**Published:** 2021-05-02

**Authors:** Rafael Brandao, Mei Qi Kwa, Yossi Yarden, Cord Brakebusch

**Affiliations:** ^1^ Biotech Research and Innovation Center (BRIC) University of Copenhagen Denmark; ^2^ Weizmann Institute of Science Rehovot Israel

**Keywords:** ACK1, development, skin tumor

## Abstract

Activated Cdc42‐associated kinase 1 (ACK1), a widely expressed nonreceptor tyrosine kinase, is often amplified in cancer and has been shown to interact with Cell division cycle 42 (Cdc42), Epidermal growth factor receptor (EGFR), and several other cancer‐relevant molecules, suggesting a possible role for ACK1 in development and tumor formation. To directly address this scenario, we generated mice lacking a functional ACK1 gene (ACK1 ko) using CRISPR genome editing. ACK1 ko mice developed normally, displayed no obvious defect in tissue maintenance, and were fertile. Primary ACK1‐null keratinocytes showed normal phosphorylation of EGFR, but a tendency toward reduced activation of AKT serine/threonine kinase 1 (Akt) and Mitogen‐activated protein kinase 1 (Erk). DMBA/TPA‐induced skin tumor formation did not reveal significant differences between ACK1 ko and control mice. Deletion of the ACK1 gene in the breast cancer cell lines MDA‐MB‐231, 67NR, MCF7, 4T1, and T47D caused no differences in growth. Furthermore, EGF‐induced phosphorylation kinetics of Erk, Akt, and p130Cas were not detectably altered in T47D cells by the loss of ACK1. Finally, loss of ACK1 in MDA‐MB‐231 and T47D breast cancer cells had a very limited or no effect on directed cell migration. These data do not support a major role for ACK1 in Cdc42 and EGFR signaling, development, or tumor formation.

## Graphical abstract

Mice with a ko of the Cdc42 effector Activated Cdc42‐associated kinase 1 (ACK1) showed normal development and normal chemically induced skin tumor formation. ACK1 ko in breast cancer cell lines did not impair proliferation or EGF signaling. These data suggest that ACK1 contrary to the expectations is lacking a major role in development or tumor formation.

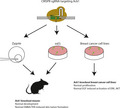

**Abbreviations**
ACK1: Activated Cdc42‐associated kinase 1
ADHD: Attention‐deficit/hyperactivity disorder
Akt: AKT serine/threonine kinase 1
Cas: Crk‐associated substrate
Cdc42: Cell division cycle 42
CRIB: Cdc42‐ and Rac‐interactive binding
CRISPR: Clustered regularly interspaced short palindromic repeats
DMBA: 7,12‐Dimethylbenz[*a*]anthracene
EGFR: Epidermal growth factor receptor
EMT: Epithelial‐to‐mesenchymal transition
Erk: Mitogen‐activated protein kinase 1
ES: Embryonic stem
GTP: Guanosine triphosphate
HRP: Horseradish peroxidase
IgG: Immunoglobulin G
MIG6: Mitogen‐inducible gene 6 protein
PCR: Polymerase chain reaction
Src: SRC proto‐oncogene, non‐receptor tyrosine kinase
STAT: Signal transducer and activator of transcription
TPA: 12‐O‐tetradecanoylphorbol‐13‐acetate

Activated Cdc42‐associated kinase 1 (ACK1; gene name *TNK2*) is an ubiquitously expressed nonreceptor tyrosine kinase (non‐RTK) that is frequently amplified and mutated in different human cancers [1]. In 488 head‐and‐neck squamous cell carcinoma analyzed by The Cancer Genome Atlas (TCGA) consortium, 59% show low‐level gain alterations of the TNK2 gene, and 13% show amplification, resulting in increased mRNA expression of Ack1 in these tumors (www.cbioportal.org). In breast cancer, activated ACK1 correlates negatively with survival [2, 3]. On the other hand, resistance of melanoma cell lines against the BRAF inhibitor vemurafenib correlated with a loss of Ack1 protein, which triggered increased expression of EGFR by a post‐translational mechanism [4].

Besides a tyrosine kinase domain, ACK1 contains a sterile α motif domain, a Src homology domain 3 (SH3), Cdc42‐ and Rac‐interactive binding (CRIB) domain‐binding Cell division cycle 42 (Cdc42), a clathrin‐interacting region, a WW domain, a Mitogen‐inducible gene 6 protein (MIG6) homology region binding to EGFR, and a ubiquitin association (UBA) domain [1]. Receptor tyrosine kinases such as EGFR, insulin receptor, Platelet‐derived growth factor receptor beta, and the neurotrophin receptor Tyrosine receptor kinase can bind to the MIG6 homology region of ACK1 and activate ACK1 by overcoming an autoinhibitory interaction. ACK1 then mediates EGF‐induced EGFR degradation, probably by binding to ubiquitin ligases via its UBA domain or by association with clathrin‐coated pits via its clathrin‐interacting domain [5]. Mutations of the UBA domain prevented EGF‐induced degradation of EGFR and ACK1, increased mitogenic signaling [6], and correlated with severe autosomal recessive infantile‐onset epilepsy [7]. In addition, ACK1 regulates trafficking of the EGFR to the p62/Next to BRCA1 gene 1 protein pre‐autophagosome [8]. Since ACK1 can be activated by different receptor tyrosine kinases (RTK), it was suggested to be a central signaling integrator for RTKs.

Cdc42 is a small GTPase regulating in particular actin cytoskeleton organization and cell polarity and active, GTP‐bound Cdc42 binds to ACK1 via the CRIB domain. This interaction was shown to be important for EGFR‐dependent activation of ACK1 [9] and ACK1/p130Cas‐dependent regulation of cell spreading [10]. In breast cancer cells, ACK1 mediates Cdc42‐dependent cell migration and signaling to p130Cas [11]. Cdc42‐mediated ACK1 activation was furthermore suggested to play a role in the endocytosis of clathrin‐coated pits [12]. In addition, Cdc42 activation stimulates ACK1‐dependent inhibition of the dopamine transporter that plays a role in Attention‐deficit/hyperactivity disorder, autism, and infantile Parkinsonism [13]. Finally, ACK1 was found to affect other Cdc42 effectors, since ACK1 phosphorylates the Cdc42 effector Wiskott–Aldrich syndrome protein, promoting its actin polymerizing activity [14]. These data suggest that ACK1 is an important Cdc42 effector molecule.

Furthermore, ACK1 was shown to directly phosphorylate AKT serine/threonine kinase 1 (Akt) at tyrosine 176 *in vitro* and *in vivo*, leading to Akt activation and pro‐survival signaling promoting tumor formation [2]. In hepatocellular cancer, ACK1 expression correlated with Akt activation and epithelial‐to‐mesenchymal transition (EMT; [15]). Moreover, ACK1 was shown to interact with a number of other molecules such as the estrogen receptor coactivator Lysine demethylase 3A, which contributes to its tumor‐promoting functions [16].

Despite the important roles suggested for ACK1 in different signaling pathways and different diseases, the *in vivo* function of ACK1 has hardly been investigated. To study ACK1 function in development and tumor, we therefore generated mice and breast cancer cell lines with a deletion of the ACK1 gene and studied the biological consequences. Contrary to the expectations, our data did not indicate an obvious role for ACK1 in development, tissue maintenance, skin tumor development, or proliferation of breast cancer cells.

## Materials and methods

### Materials

Unless specified otherwise, chemicals were purchased from Merck. Water was double‐distilled and purified using Milli‐Q system (Millipore, Søborg, Denmark). PBS used in this study was prepared and autoclaved by laboratory facilities. Pipettes, test tubes, plates, and dishes were obtained from Greiner (Frickenhausen, Germany).

### Animals

Animals were kept according to national and European animal welfare laws in an Association for Assessment and Accreditation of Laboratory Animal Care‐accredited animal house under SPF conditions. Tumor experiments were approved by the Danish Board for Animal Experiments (Dyrefosøgstilsynet). For all experiments, littermate controls were used.

### Generation of ACK1 ko mice

The target sequence for the murine ACK1 gene was selected using the website crispr.mit.edu, prioritizing for location close to the translation start and low predicted off‐target effects. Double‐stranded oligonucleotides containing the target region were then cloned into pX330 (Addgene #42230; Addgene, Watertown, MA, USA) following published protocols [17].

ACK1 ko mice were generated by direct injection of Cas9 mRNA and sgRNA prepared from the pX330 vector targeting ACK1 into morulas derived from B6/N mice or by transient co‐transfection of mouse Embryonic stem (ES) cells (TCF2.2; hybrid 129S2/C57BL/6N; [18]) with the pX330 vector targeting ACK1 and a puromycin resistance expression vector, followed by morula injection. Recombinants were identified by sequencing of a 250 bp genomic Polymerase chain reaction (PCR) fragment containing the target sequence (ACK1‐gCheck‐forward: 5′‐GGAGAGGGTCACCTGGTC; ACK1‐gCheck‐reverse: 5′‐CACCCTGTGAACAGCACTC).

### Genotyping of mice

Mice carrying a wild‐type ACK1 allele were identified by genomic PCR with touchdown protocol with a final annealing temperature of 59 °C using the primers ACK1‐forward (GGAGAGGGTCACCTGGTC) and ACK1‐wild‐type‐reverse (GTTCCCTCCTCCGGCT).

Mice carrying a 5Δ ACK1 allele were identified by genomic touchdown protocol with a final annealing temperature of 59 °C using the primers ACK1‐forward and ACK1‐5∆‐reverse (AGCCCGTTCCCTCCCT). Mice carrying an allele with a 4‐nucleotide insertion were identified by genomic touchdown protocol with an annealing temperature of 63 °C using ACK1‐forward and ACK1‐I4‐reverse (CCGTTCCCTCCTCCCC) primers.

### Skin tumor formation

Skin tumor formation was induced in 8‐week‐old female mice by single treatment with 7,12‐dimethylbenz[*a*]anthracene (DMBA) and repeated treatment with 12‐O‐tetradecanoylphorbol‐13‐acetate (TPA) as described before [19].

### Histology

Paraffin sections of mouse skin fixed with 4% paraformaldehyde in PBS were stained by hematoxylin/eosin (H/E) following standard protocols.

### Cell culture

Primary mouse keratinocytes were isolated and cultured as described earlier [20].

MDA‐MB‐231 (ATCC CRM‐HTB‐26; Manassas, VA, USA), 4T1 (ATCC CRL‐2539), MCF7 (ATCC HTB‐22), 67NR (ExPASy CVCL 9723), and T47D (ATCC HTB‐133) breast cancer cells were obtained from other laboratories and cultured in Dulbecco's Modified Eagle Medium (Thermo Fisher Scientific, Lillerød, Denmark), 10% FBS (Thermo Fisher Scientific, HyClone, #SV30180.03), 100 U·mL^−1^ penicillin/streptomycin (Thermo Fisher Scientific, Gibco, #15140‐122) at 37 °C with 5% CO_2_.

### Generation of ACK1 ko breast cancer cell lines

The target sequence for the human ACK1 gene was designed using the CRISPR design software (crispr.mit.edu) and selected for location close to the translation start and low predicted off‐target effects. Double‐stranded oligonucleotides containing the target region were then cloned into the puromycin resistance containing lentiviral CRISPR vector lentiCRISPR v2 (Addgene #52961) following published protocols [19]. Lentivirus was generated using the packaging plasmids psPAX2 (Addgene #12260) and pCMV‐VSV‐G (Addgene #8454). Transduction and puromycin selection of transduced breast cancer cells was carried out following standard protocols. Control cells were transduced with the empty lentiCRISPR v2 vector. Genotyping was carried out as described above. Sequencing data of the polyclonal cell mixtures were analyzed using the TIDE decomposition tool (www.shinyapps.datacurators.nl/tide/).

### Proliferation assay

Fifty thousand cells were seeded per well of a 12‐well plate in triplicates. During 5 days, each day one set of cells was washed once with PBS, fixed in 500 µL of 4% PFA for 20 min at room temperature (RT), and stained with 500 µL of crystal violet (Sigma, Søborg, Denmark, #C6158) diluted in 20% methanol/PBS for 15 min at RT. Cells were then washed in PBS followed by wash in running water and dried overnight. After the last plate been fixed and stained, 500 µL of 1% SDS in PBS was added to each well. Hundred microlitre from each well was transferred to a 96‐well plate, and absorbance was measured at 562 nm using an ELISA plate reader.

### SDS/PAGE and western blotting

Cultured cells were lysed with ice‐cold RIPA buffer containing protease and phosphatase inhibitors. Lysates were separated by a 10% SDS/PAGE, and western blotting was carried out following standard protocols. For detection, the following primary antibodies were used: ACK1 (Santa Cruz, Santa Cruz, CA, USA, #28336), E‐cadherin (Invitrogen, Carlsbad, CA, USA, #131900), EGFR (#2232), Erk (#91025), pAkt (S483) (#9271), pEGFR (Y1068) (#3777), pErk (T202/Y204) (#9101), pp130Cas (Y410) (#4011; all Cell Signaling, Danvers, MA, USA), keratin‐14 (Covance, Copenhagen, Denmark, PRB‐155P), p130Cas (Becton Dickinson, Lyngby, Denmark, 610272), and β‐actin (Abcam, Cambridge, MA, USA, #AB6276). The loading control was always checked on the blot of the corresponding sample. If different primary antibodies were used for the same blot membrane, the same loading control was used.

For detection, appropriate Horseradish peroxidase (HRP)‐coupled secondary antibodies were used: horse anti‐mouse Immunoglobulin G (IgG; #VECTPI2000), goat anti‐rabbit IgG (#VECTPI1000, all Vector Laboratories, Burlingame, CA, USA), and donkey anti‐rat (Jackson Immunoresearch, Cambridge, UK, #712‐035‐153).

Luminata™ Western HRP Chemiluminescence Substrates detection reagent (Millipore) was used for chemiluminescence, which was then detected with Medical X‐Ray film (AGFA, Birkerød, Denmark). The intensity of the bands was quantified using imagej software.

### Cell migration assay

Control and ACK knockout T47D or MDA‐MB‐231 cells (1 × 10^4^ cells) were grown for 16 h overnight in 12‐well tissue culture plates. Thereafter, the plates were placed in the Incucyte ZOOM (Essen Bioscience) and time‐lapse microscopy was performed to monitor the movement of the cells at 20‐min intervals for a total of 8 h. Migration of individual cells was tracked manually using the imagej software. Cell migration trajectory, velocity, and mean‐squared displacement (MSD) were assessed using the open‐source computer software DiPer [21]. An average of 50 cells per sample were assessed.

### Statistical analysis

Error bars indicate SD or confidence interval (CI), as indicated. Statistical significance was measured using T‐test SciPy Python package (ns *P* > 0.05; *P ≤ 0.05; **P ≤ 0.01; ***P ≤ 0.001).

## Results

### Normal developmental of ACK1 ko mice

ACK1 is suggested to be an important regulator of EGFR and Cdc42 signaling, which both have crucial roles in development and tissue maintenance.

**Fig. 1:**
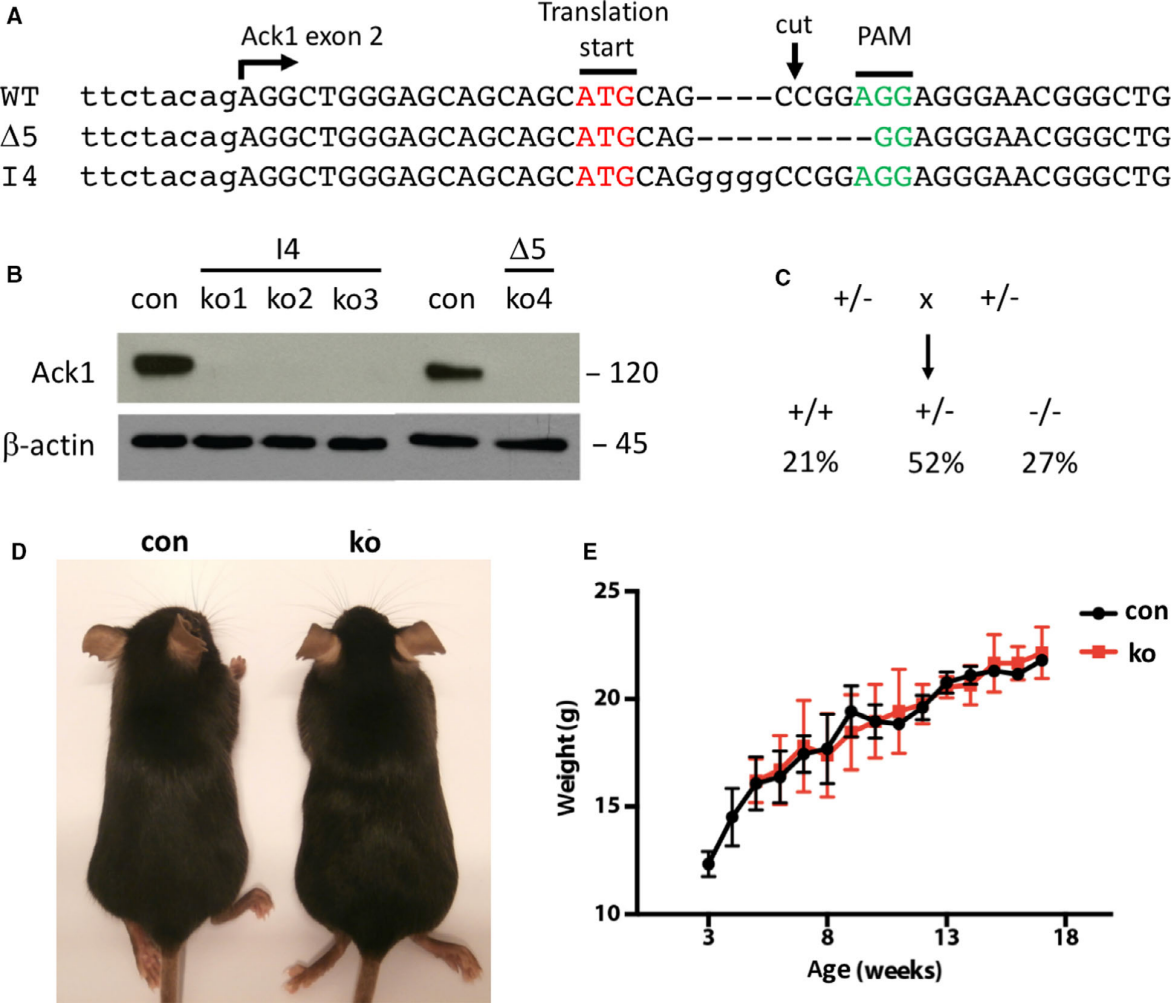
ACK1 is dispensable for development. (A) Sequence of wild‐type (WT) and ACK1 ko mice (Δ5, I4) at the start of the ACK1 gene. Indicated are the start of exon 2, the translation start site (ATG), the cut site of the Cas9/gRNA, and the protospacer adjacent motif (PAM). (B) Western blot of lysates of primary keratinocytes isolated from control (con) and ACK1 ko mice for ACK1 and β‐actin. (C) Genotyping of offspring of heterozygous ACK1 ko mice (+/−) indicating Mendelian distribution (*n*: 51). (D) Representative picture of 8‐week‐old female control and ACK1 ko mice, not displaying any obvious morphological differences. (E) Weight curve of female control and ACK1 ko mice, not displaying a significant difference (*n*: 5/5; error bars are SD).

To generate mice with a functional inactivation of the ACK1 encoding *Tnk2* gene by CRISPR/Cas9 genome editing, an sgRNA was designed inducing a double‐stranded DNA cut 4 bp downstream of the start codon (Fig. 1A). ES cells were transfected with a vector expressing Cas9 and sgRNA, resulting in point mutations of the ACK1 gene in approx. 50% of the clones analyzed. An ES cell clone with a 5 bp frameshift deletion (5∆) downstream of the ACK1 start codon (Fig. 1A) was then injected into morula, giving rise to highly chimeric germline transmitting mice. In parallel, Cas9 mRNA and sgRNA were directly injected into mouse zygotes, giving rise to 13 pups, of which two showed a mutation in the ACK1 gene. A line with a 4 bp frameshift insertion (I4; Fig. 1A) was chosen for further experiments. Sequencing of the four most likely off‐target sites predicted by the CRISPR design software did not reveal any mutations in 5Δ ES cells or I4 mice (data not shown).

Both, the Ι4 and the Δ5 mouse lines were bred to homozygosity and then tested for the absence of ACK1 protein. Western blot analysis of primary keratinocytes isolated from the skin of both lines with an antibody raised against a C‐terminal peptide of ACK1 showed complete loss of the protein, while keratinocytes from wild‐type littermates showed strong expression of ACK1 (Fig. 1B).

ACK1 ko mice were born at normal Mendelian ratio (Fig. 1C) and showed normal growth (Fig. 1D,E) and cage behavior (body posture, exploration, nesting, huddling, grooming). By visual appearance, ACK1 knockout mice were indistinguishable from their control counterparts at least up to 6 months of age, which were the oldest mice kept. Male and female ACK1 ko mice were fertile and gave rise to normal litter sizes (data not shown).

These data do not indicate any obvious developmental phenotype of ACK1 ko mice and show no difference between the I4 and the Δ5 ACK1 ko mice. Further analysis was therefore restricted to the Δ5 mouse strain.

### Normal skin organization in ACK1 ko mice

**Fig. 2:**
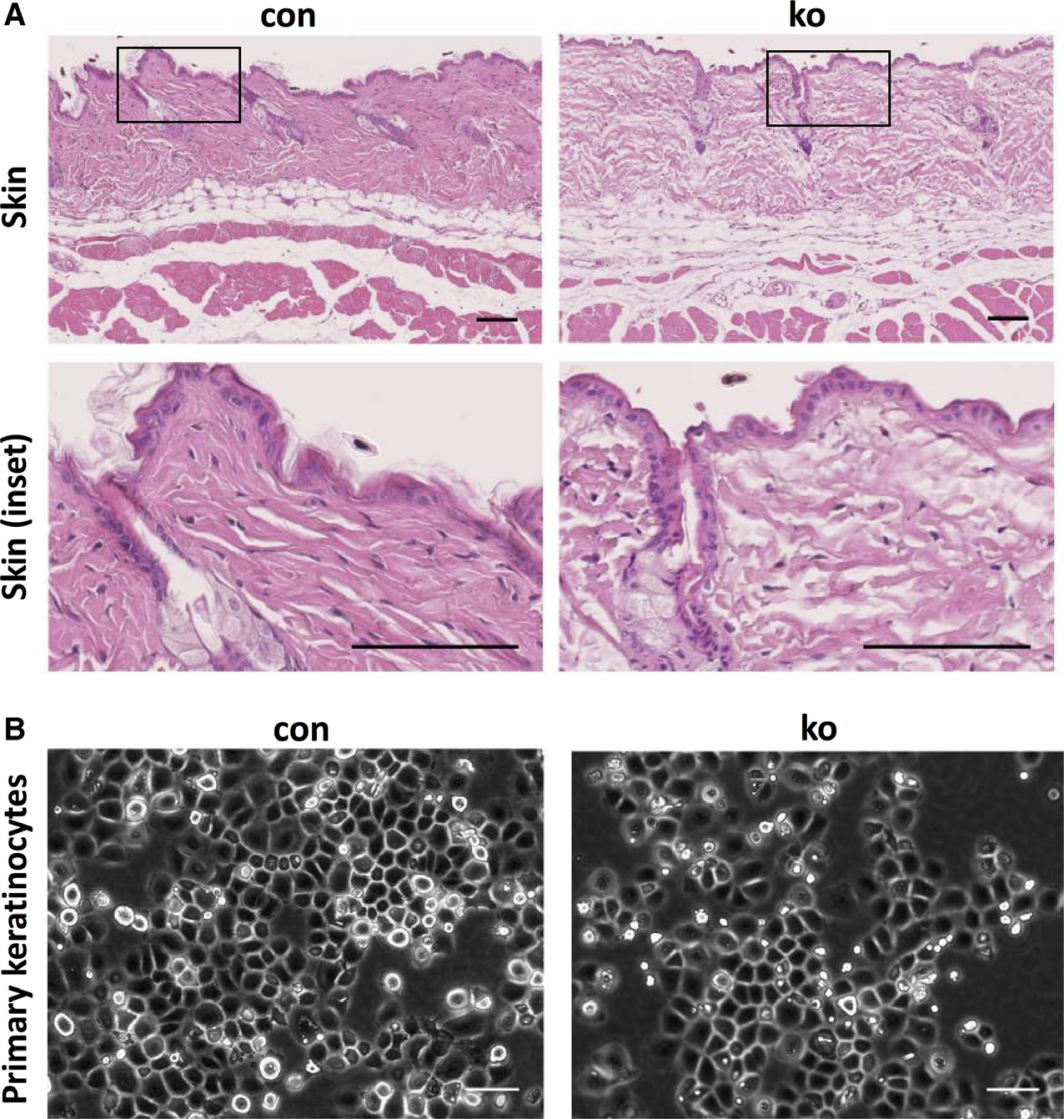
Normal skin of ACK1 ko mice. (A) Hematoxylin/eosin‐stained skin sections of 8‐week‐old control and ACK1 ko mice (bar = 100 µm; *n*: 3/3). (B) Cultured primary keratinocytes isolated from control and ACK1 ko mice (bar = 100 µm).

Histological analysis of the skin of 8‐week‐old control and ACK1 ko mice indicated normal epidermal organization and thickness (Fig. 2A). Hair follicles in the resting stage with clearly distinguishable sebaceous glands were found in all mice tested (Fig. 2A). Dermis, subcutaneous fact, and underlying muscle layer were similar in control and ko mice. No inflammatory infiltrate was observed in any of the skin sections analyzed. Primary keratinocytes isolated from control and ko mice showed similar cell area and growth with tightly compacted islands of epithelial‐looking cells with typical ‘cobblestone’ appearance (Fig. 2B).

Based on these results, ACK1 appears to be dispensable for skin development and maintenance.

### Reduced EGF‐induced Akt phosphorylation in ACK1‐null keratinocytes

ACK1 was reported to regulate EGF‐induced reduction in EGFR, mitogenic signaling, activation of Akt, phosphorylation of the scaffold protein p130Cas, and EMT. We therefore tested whether these pathways are altered in primary keratinocytes lacking ACK1 by overnight starvation with serum‐free medium and stimulation with 100 ng·mL^−1^ EGF for 30 min.

**Fig. 3:**
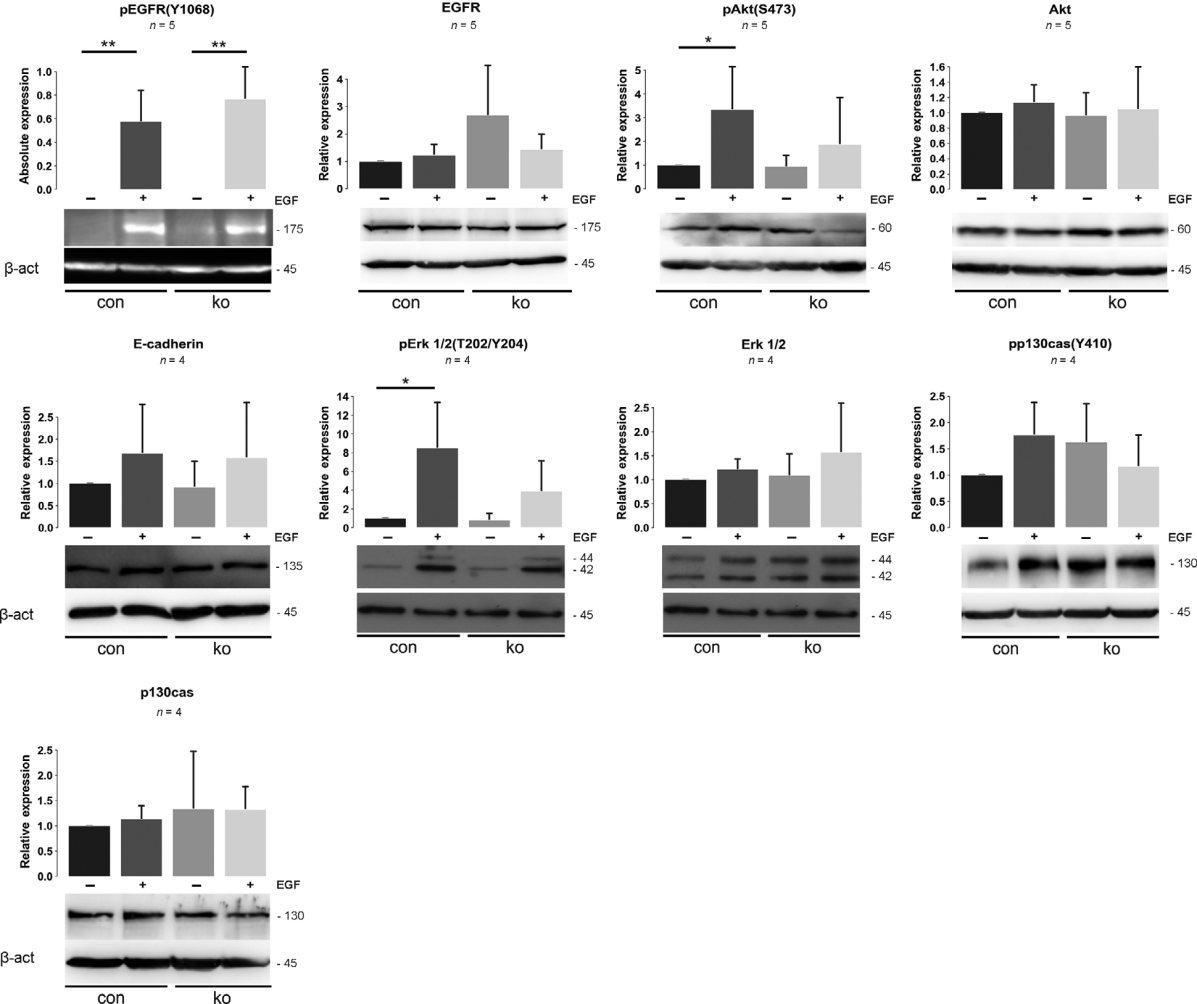
Altered EGFR turnover in ACK1 ko keratinocytes. Western blot analysis of lysates of primary keratinocytes of control and ACK1 ko mice, stimulated or not stimulated with EGF, for indicated proteins, with corresponding quantifications (*n*: 4‐5; CI: 95%; one‐way ANOVA, *P ≤ 0.05; **P ≤ 0.01; the following primary antibodies were tested on the same blot membrane and share the loading controls: (pEGFR, Akt), (pErk1/2, Erk1/2)).

EGF treatment induced a strong increase in tyrosine phosphorylation of the EGFR (Tyr1068) in both control and ACK1 ko cells, indicating activation of the EGFR (Fig. 3). No significant changes were observed for EGFR in control and ko cells, neither in the presence nor in the absence of EGF. These data do not suggest an essential role of ACK1 in EGFR activation in primary keratinocytes.

EGF stimulation induced phosphorylation of Erk1/2 (T202/Y204) and of Akt (S473) (Fig. 3). These changes were significant in control, but not in ko keratinocytes, suggesting that ACK1 might contribute to Erk and Akt activation downstream of EGFR in keratinocytes. p130Cas was reported to be phosphorylated by EGF stimulation (pp130cas(Y410)), but no significant changes were detectable in primary keratinocytes treated for 30 min with EGF (Fig. 3). Total levels of Erk1/2, Akt, and p130Cas were not altered (Fig. 3).

Finally, we tested whether loss of ACK1 decreases E‐cadherin expression, since ACK1 was reported to control EMT. However, no significant change in E‐cadherin could be observed in control and ACK1 ko keratinocytes in the presence and absence of EGF (Fig. 3).

These data suggest that ACK1 is not crucial for EGFR activation in keratinocytes, although a subtle role cannot be excluded.

### ACK1 is not required for skin tumor formation

**Fig. 4:**
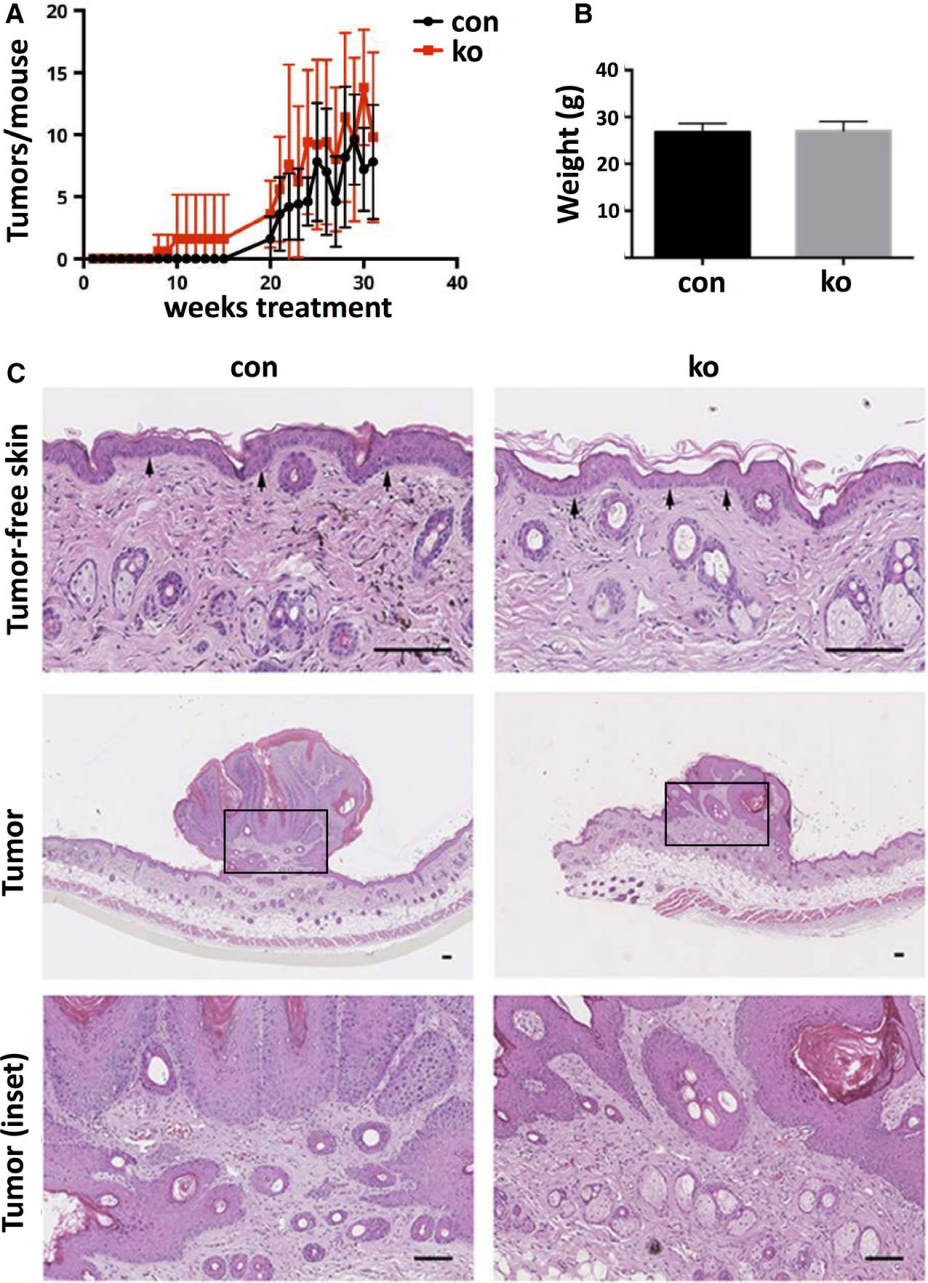
No obvious alteration in DMBA/TPA‐induced skin tumor development in ACK1 ko mice. (A) Frequency of tumors with a diameter of more than 1 mm in control and ACK1 ko mice treated with DMBA/TPA (*n*: 5/5; error bars are SD). (B) Body weight of control and ACK1 ko mice at the end of the DMBA/TPA treatment (*n*: 5/5; error bars are SD). (C) Hematoxylin/eosin‐stained skin sections of control and ACK1 ko mice at the end of the DMBA/TPA treatment (bar = 100 µm; *n*: 3/3).

Since ACK1 expression is increased in many cancer types, we investigated whether ACK1 is important for DMBA/TPA‐induced skin tumor formation, where the back skin of mice is treated once with the mutagen DMBA and then twice a week with the tumor promoter TPA. Testing five control and five ACK1 ko mice, we could not detect a significant difference in onset of tumor formation or tumor frequency per mouse (Fig. 4A). Body weight of the mice at the end of the experiment was similar (Fig. 4B), suggesting no adverse effects of the treatment in addition to skin tumor formation.

Histological analysis of back skin treated with DMBA/TPA revealed comparable hyperplasia in the tumor‐free epidermis of control and ACK1 ko mice (Fig. 4C). Skin tumors showed exophytic growth, normal layered organization and terminal differentiation, absence of dysplasia, and a sharply demarcated border between the neoplastic epithelium and the underlying dermis, suggesting the formation of benign skin tumors in both control and ACK1 ko mice (Fig. 4C).

These data do not support an essential role for ACK1 in skin tumor development.

### Loss of ACK1 does not interfere with tumor‐relevant signaling pathways

Next, tumors and DMBA/TPA‐treated tumor‐free skin were analyzed for ACK1‐ and tumor‐relevant signaling pathways.

**Fig. 5:**
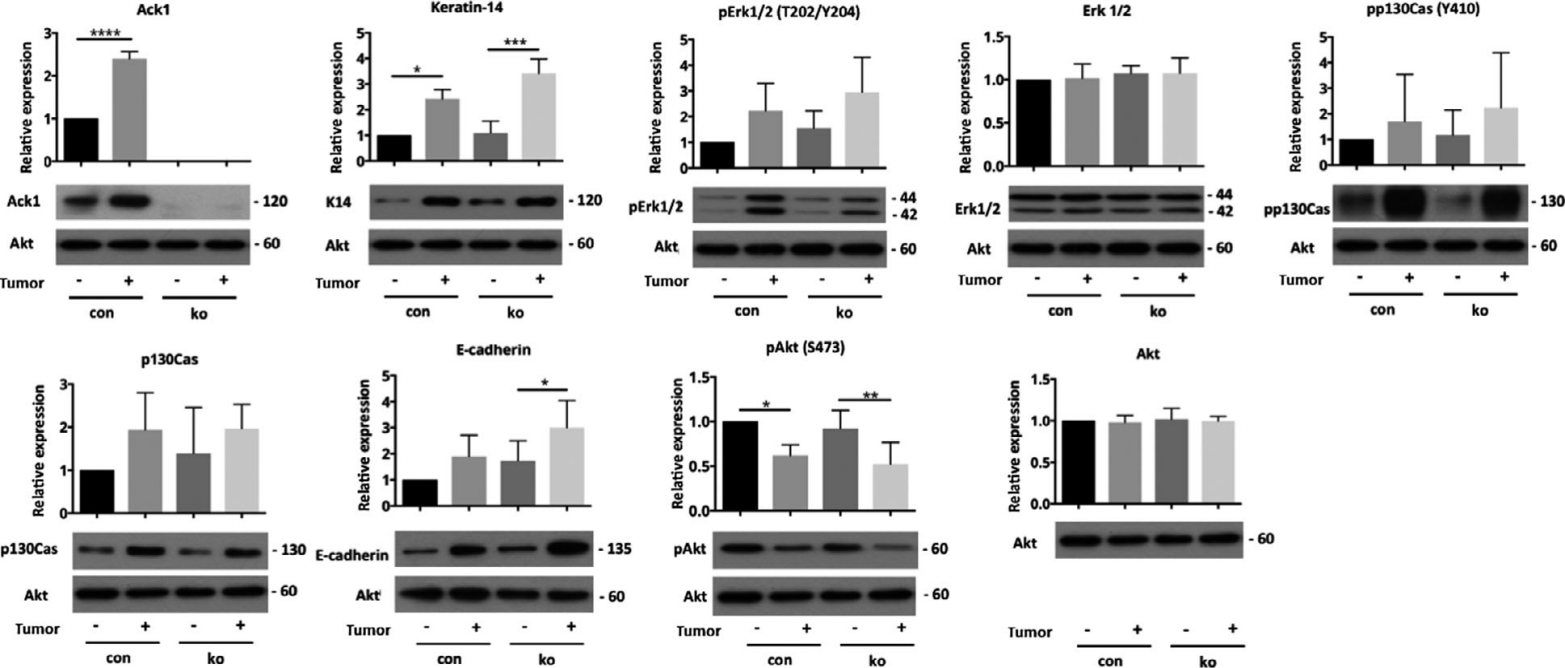
Unaltered signaling in skin tumors of ACK1 ko mice. Western blot analysis of lysates skin tumors and tumor‐free skin of DMBA/TPA‐treated control and ACK1 ko mice for indicated proteins, with corresponding quantifications. Similar amount of proteins were applied per lane. Akt was used as loading control, since β‐actin was found to vary between tumors and tumor‐free skin (*n*: 3–5/3–5; error bars are standard deviation; one‐way ANOVA, *P ≤ 0.05; **P ≤ 0.01; ***P ≤ 0.001; **** to *P* ≤ 0.0001; The following primary antibodies were tested on the same blot membrane and share the loading control: (Ack1, keratin‐14, pp130Cas), (pErk1/2, Erk1/2), (p130Cas, pAkt, and Akt).

Expression of ACK1 was increased in tumors compared with tumor‐free skin of control mice, while, as expected, no ACK1 was detected in ACK1 ko tumors and tumor‐free skin (Fig. 5). Tumors of control and ACK1 ko mice showed increased expression of the basal keratinocyte marker keratin‐14 (K14), which might be due to an increased percentage of basal keratinocytes in the tumors compared with tumor‐free skin (Fig. 5). Similar to K14, also the expression of pErk, pp130Cas, and E‐cadherin was increased in tumor compared with tumor‐free skin, although these changes were mostly not significant (Fig. 5). No obvious difference was observed between control and ACK1 ko mice. pAkt was decreased in tumors compared with tumor‐free skin, in both control and ACK1 ko mice (Fig. 5). This might be related to the known increase in pAkt in suprabasal keratinocytes (Wang *et al*., 2010) and the relative decrease in K14‐negative suprabasal cells in the tumor samples. pAkt levels were similar in control and ACK1 ko mice. Total levels of Erk and Akt were unchanged tumor and tumor‐free tissues and control and ACK1 ko mice, while p130Cas showed a tendency to be increased in tumors (Fig. 5).

These data do not indicate an important role for ACK1 in the regulation of mitogenic (pErk) or pro‐survival (pAkt) signaling, EMT (E‐cadherin), or p130Cas‐dependent migration in hyperplastic skin or benign skin tumors.

### ACK1‐null breast cancer cell lines show no obvious changes in proliferation or EGFR signaling

**Fig. 6:**
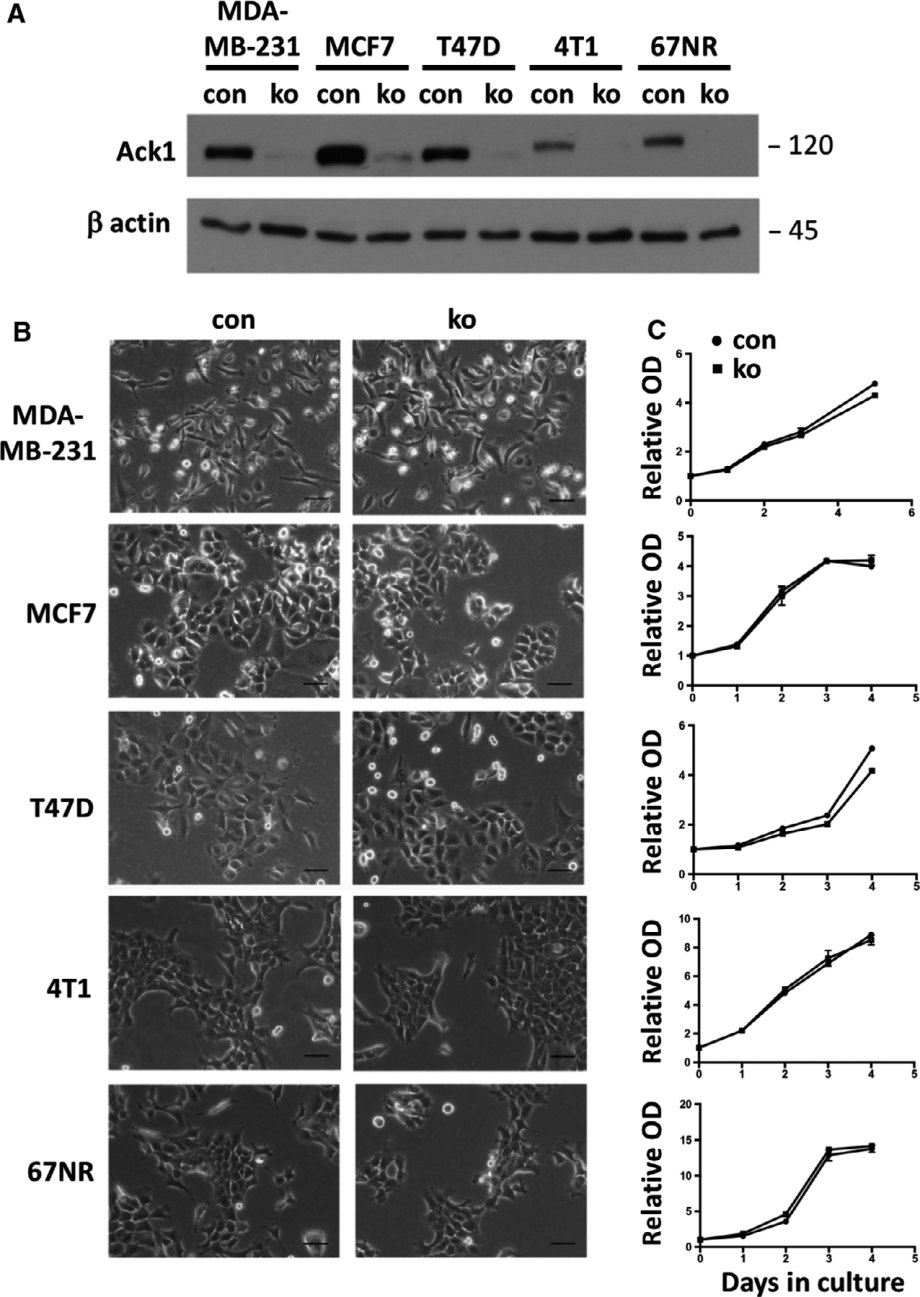
Normal morphology and growth of breast cancer cell lines lacking ACK1. (A) Western blot of lysates of indicated breast cancer cells with (con) and without (ko) ACK1 for ACK1 and β‐actin, indicating efficient deletion of the ACK1 gene. (B, C) Morphology and growth (OD: optical density in crystal violet assay) of indicated breast cancer cells with (con) and without (ko) ACK1 (bar= 100 µm; *n*: 3/3).

To test the role of ACK1 in malignant cancer cells, we chose two breast cancer cell lines of the basal type (MDA‐MB‐231 and 4T1) and three of the luminal type (MCF7, T47D, and 67NR). All breast cancer cell lines expressed ACK1 (Fig. 6A), but there was no obvious correlation of the ACK1 level with the breast cancer subtype or reported invasiveness. The ACK1 gene was deleted by CRISPR genome editing using lentiviral transduction. Transduced cells were enriched by antibiotic selection and analyzed without subcloning. Efficient targeted mutation was confirmed by sequencing of genomic PCR fragments, where TIDE analysis of the polyclonal sequences revealed that nearly all cells showed frameshift mutations (data not shown). Western blot analysis confirmed very efficient loss of ACK1 protein in the ko cells (Fig. 6A).

Deletion of the ACK1 gene did not affect significantly morphology and *in vitro* growth of the cancer cell lines (Fig. 6B,C), indicating that ACK1 is not a major factor determining proliferation of these breast cancer cell lines. Furthermore, cell morphologies were not detectably changed.

**Fig. 7:**
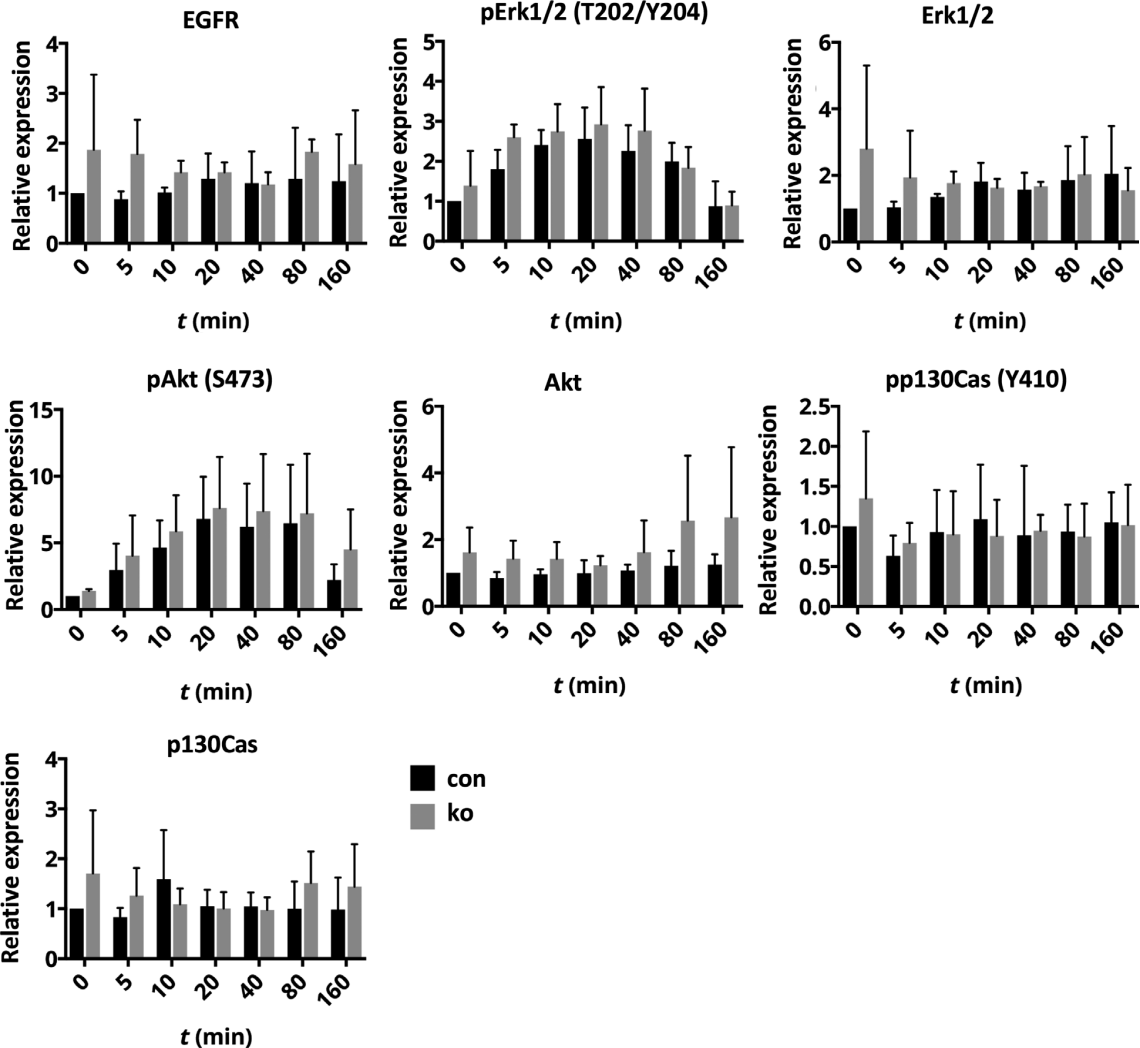
Normal EGFR signaling in the breast cancer cell line T47D lacking Ack. Quantification of western blots for indicated proteins of lysates of T47D breast cancer cells with (con) or without (ko) ACK1, stimulated with EGF for indicated times, for, with corresponding quantifications (*n*: 3/3; error bars are SD).

To identify more subtle alterations of signaling pathways, we performed an EGF stimulation kinetic in T47D cells. T47D cells showed strong induction of phosphorylated forms of Erk and Akt, but not of p130Cas (Figs 7 and S1). Total levels of these proteins were not altered. Loss of ACK1 did not result in an obvious change in these kinetic changes. In starved MDA‐MB‐231 cells with or without, EGF treatment did not result in significant changes in EGFR, pErk, Erk, pAkt, Akt, ppCas130, and pCas130 (Figs S2,S3).

These data do not indicate an important role of ACK1 for EGFR signaling in breast cancer cells.

### ACK1 might be of minor importance for the migration of breast cancer cells

**Fig. 8:**
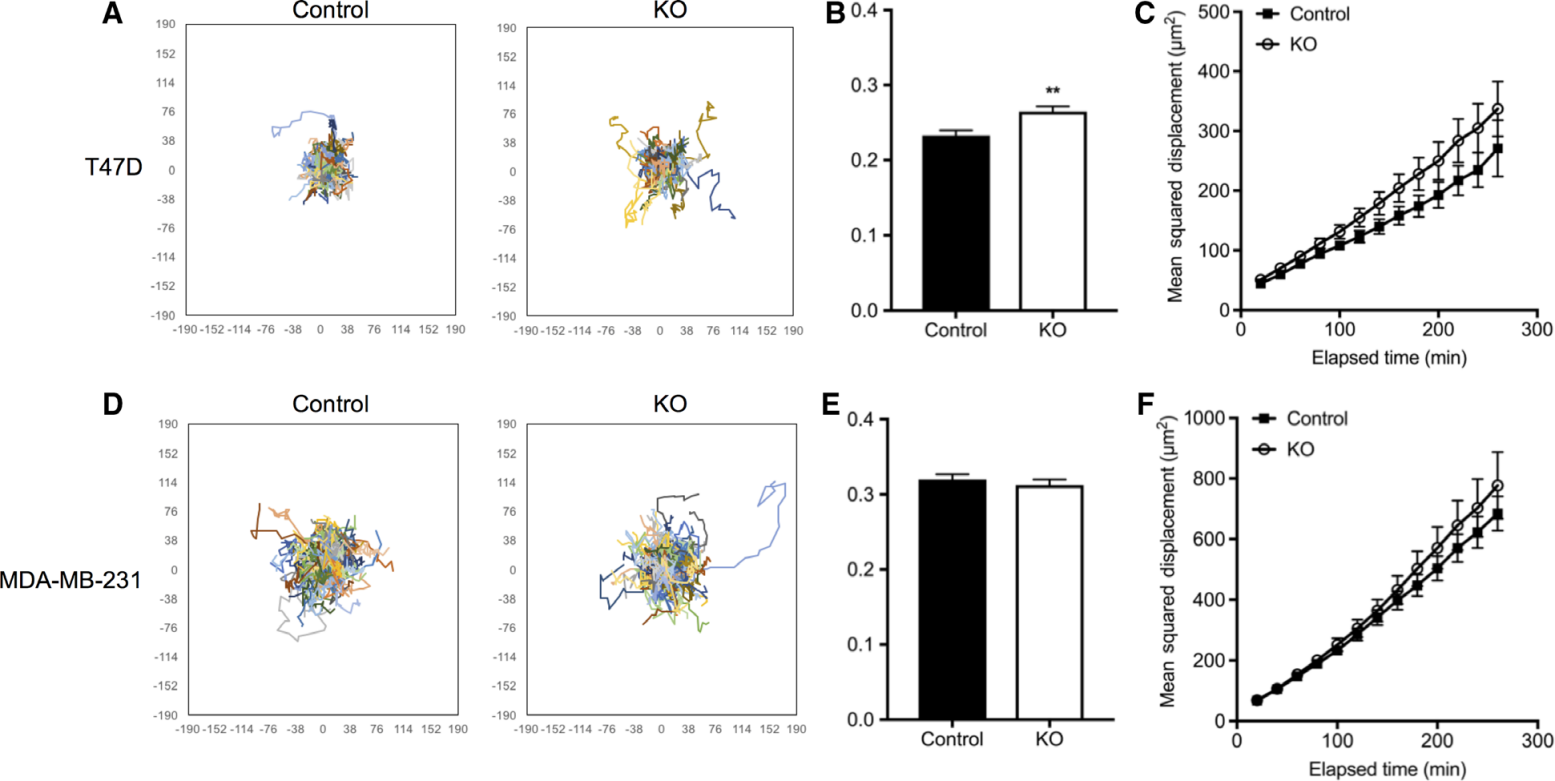
Migration of MDA‐MB‐231 and T47D breast cancer cells lacking ACK1 is hardly altered. Overlaid migration paths (A), average migration speed (B), and mean square displacement (MSD; C) of MDA‐MB‐231 and T47D breast cancer cells (D, E, and F, respectively) with (con) or without (ko) ACK1 during 8 h (*n*: 3/3; error bars are SD; one‐way ANOVA, ** *P* ≤ 0.01).

To test the role of ACK1 in breast cancer cell migration, we analyzed movement of MDA‐MB‐231 and T47D cells with and without ACK1 by time‐lapse migration movies. Single‐cell migration was evaluated using imagej for migration speed and mean square displacement, which combines speed and directional persistence. In general, MDA‐MB‐231 showed higher migration speed than T47D cells (Fig. 8). In both cell lines, mean square displacement of ACK1 ko cells was slightly lower than in controls, although these changes were not significant with the number of experiments carried out. With respect to migration speed, ACK1 ko T47D cells showed a slightly higher speed than control cells, while no significant difference could be detected between ACK1 ko and control MDA‐MB‐231 cells.

## Discussion

ACK1 was suggested to be important for mediating biological effects of RTKs and Cdc42. ACK1 expression was found to correlate with proliferation and invasion of breast cancer cells [3], and to promote cancer progression by multiple mechanisms including activation of Akt [2], alteration of epigenetic marks [22], and inhibition of tumor suppressors [23]. These studies were done with the overexpression of ACK1, siRNA‐mediated knockdown, and small molecular weight inhibitors of ACK1. We used now a genetic system to completely eliminate ACK1 function and to study the corresponding biological consequences in mice and in breast cancer cells.

Surprisingly, mice lacking ACK1 showed normal development and normal cage behavior, and were fertile. ACK1 is expressed in keratinocytes, but no alterations in skin histology were detected in ACK1‐deficient mice. ACK1‐null keratinocytes showed no significant change in total EGFR levels and normal EGFR phosphorylation, although EGF‐induced activation of Erk1/2 and Akt might be reduced. These *in vitro* data do not support a central role for ACK1 in EGFR or RTK signaling. This notion is supported by the absence of an obvious developmental phenotype as described for mice with ko of EGFR [24]. The phenotype of ACK1 ko mice is also different from mice with a constitutive or tissue‐specific ko of Cdc42 [25], suggesting that ACK1 is not a crucial effector of Cdc42. ACK1 is expressed constitutively in the brain, but the absence of obvious behavioral alterations suggests also here only a subtle role, maybe restricted to pathological situations.

The absence of an obvious developmental phenotype could be related to overlapping functions of other nonreceptor tyrosine kinases. Testing the expression level of other nonreceptor tyrosine kinases will assess whether the loss of ACK1 is maybe compensated by the increased expression of other tyrosine kinases. The tyrosine kinase structurally most closely related to ACK1 is Tnk1/Kos1. In contrast to ACK1, however, Tnk1 is believed to have tumor suppressor function by interfering with Ras signaling [26], making it an unlikely candidate for functional redundancy with ACK1 in respect to oncogenic signaling pathways.

CRISPR genome editing can cause off‐target effects by introducing mutations at other genomic sites [27]. We could not detect such mutations at predicted off‐target sites, suggesting that frequency of off‐target reactions is low if the target sequences are chosen carefully. Furthermore, off‐target mutations should be quickly lost during breeding if they are not occurring on the same chromosome. Finally, it is very unlikely that an off‐target reaction is rescuing a phenotype. It rather would make a phenotype more complex.

In order to study skin tumor formation in ACK1 ko mice, we used the classical DMBA/TPA model. Compared to tumor‐free, DMBA/TPA‐treated skin of control mice, ACK1 levels were increased in tumors of control mice, which might correspond with increased number of proliferating, K14+ keratinocytes in the tumors. Loss of ACK1, however, did not significantly affect tumor frequency, onset, and histology. Moreover, no changes in phosphorylation of Erk, Akt, or p130Cas, or in the amounts of E‐cadherin were detected in tumor tissues or tumor‐free skin. These biochemical data corroborate our notion that ACK1 is not playing a major role in skin tumor formation.

In the DMBA/TPA skin tumor model, ACK1 ko mice showed no significant changes with respect to tumor formation and activation of signaling pathways, although ACK1 protein was increased in DMBA/TPA‐treated skin of control mice.

A dispensable role for ACK1 in a DMBA/TPA skin tumor model with wild‐type mice does not exclude a role for ACK1 in skin tumors with amplification of the ACK1 gene or in tumors of other tissues. We therefore investigated the consequences of ACK1 gene deletion on different breast cancer lines, expressing different amounts of ACK1. Ablation of ACK1 resulted neither in low nor in high expressing breast cancer cells in a detectable change in cell growth described earlier [3]. In addition, we could not detect any change in basal or EGF‐induced activation of EGFR, Erk, and Akt, and in phosphorylation of p130Cas [11]. However, migration experiments hinted a possible promoting role for ACK1 in breast cancer cell migration.

This study is not indicating a pivotal role for ACK1 in skin tumor, breast cancer, or signaling pathways shown earlier to be regulated by ACK1 in cell lines. Overexpression effects, off‐target effects of siRNA, or small molecular weight inhibitors were used, and different model systems applied might contribute to different observations in earlier reports. However, also the gene deletion model used in this study has shortcomings, as the permanent and complete loss of ACK1 protein could result in compensatory changes different to acute and partial inhibition models, which might ameliorate the phenotype [28]. Furthermore, an absent loss‐of‐function phenotype does not exclude a phenotype caused by ACK1 overexpression models, which in fact is similar to the increased expression of ACK1 observed in many tumors. In addition, while ACK1 was not essential for DMBA/TPA‐induced skin tumor, it might be of importance in other cancer models and other diseases. For example, a single nucleotide polymorphism in the ACK1 gene was found to associate with the therapy outcome in patients infected with hepatitis C virus, suggesting a possible role for ACK1 in viral infection [29]. Obviously, our data also do not contradict any reported ACK1 functions not tested in this study, such as a marker for apoptosis or activator of STAT1/STAT3 signaling [30, 31].

The lack of an obvious developmental phenotype of ACK1 ko mice suggests, on the other hand, that side effects of specific ACK1 inhibitors will be probably mild, resulting in a wide therapeutic window.

## Conflict of interest

The authors declare no conflict of interest.

## Author contributions

RB and MQK acquired and analyzed the data, YY provided crucial advice and training, CB designed the project, and RB and CB wrote the manuscript.

## Supporting information


**Fig. S1**. Representative Western blots for Fig 7.

**Fig. S2**. Normal EGFR signaling in the breast cancer cell line MDA‐MB‐231 lacking Ack. Quantification of Western blots for indicated proteins of lysates of MDA‐MB‐231 breast cancer cells with (con) or without (ko) ACK1, stimulated with EGF for indicated times, for, with corresponding quantifications (*n*: 3/3).

**Fig. S3**. Representative Western blots for Fig. S2

## Data Availability

The data that support the findings of this study are available in the figures and the [Link], [Link], [Link] of this article.
