# Supplementary figures and images for: ACK1 is dispensable for development, skin tumor formation, and breast cancer cell proliferation
Source: FEBS Open Bio. 2021 May 2;11(6):1579–92. doi: 10.1002/2211-5463.13149 (PMC8167857; doi:10.1002/2211-5463.13149)

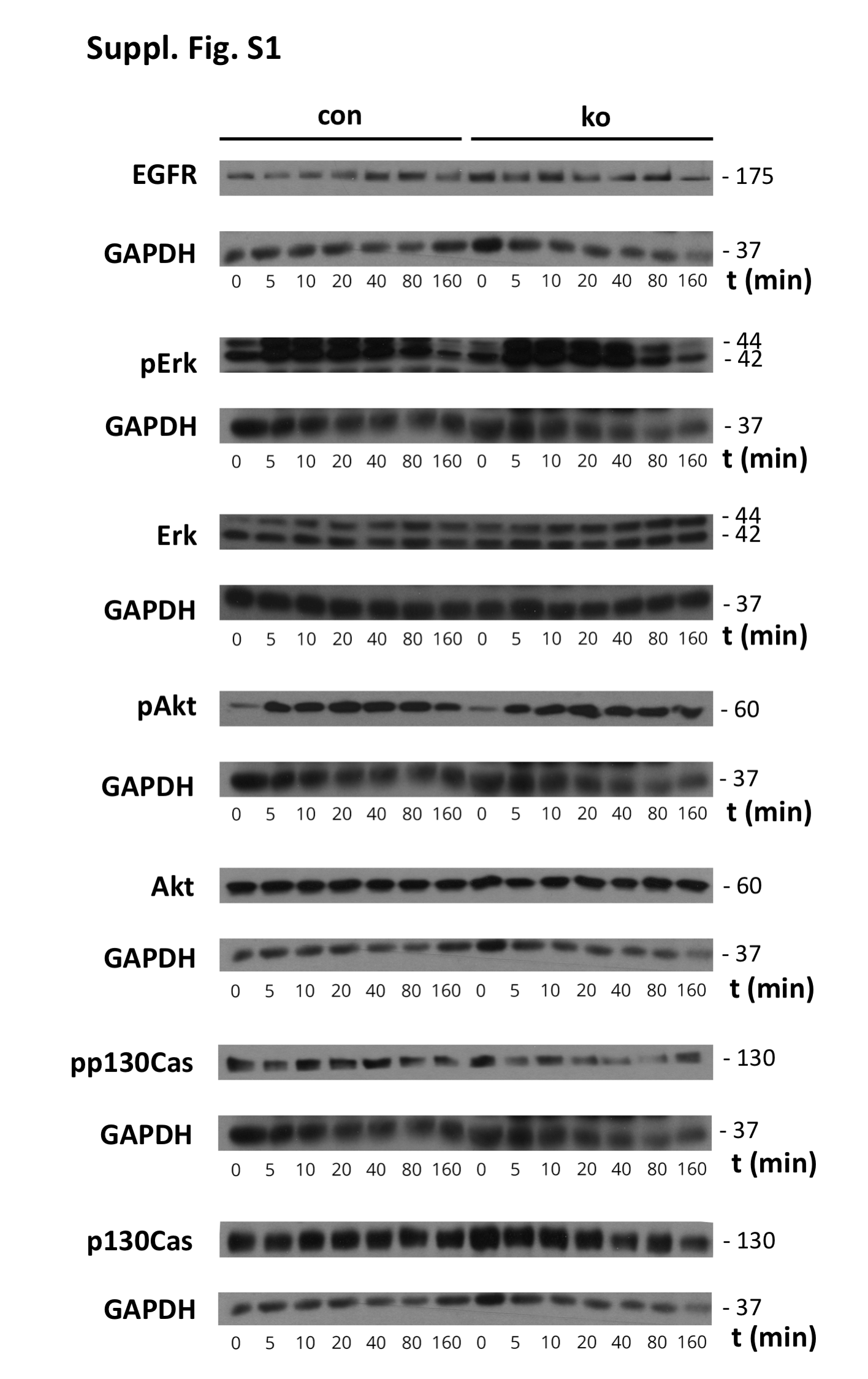

Supplement: Supplementary file 1 — Fig. S1. Representative Western blots for Fig 7. [file FEB4-11-1579-s003.tiff]

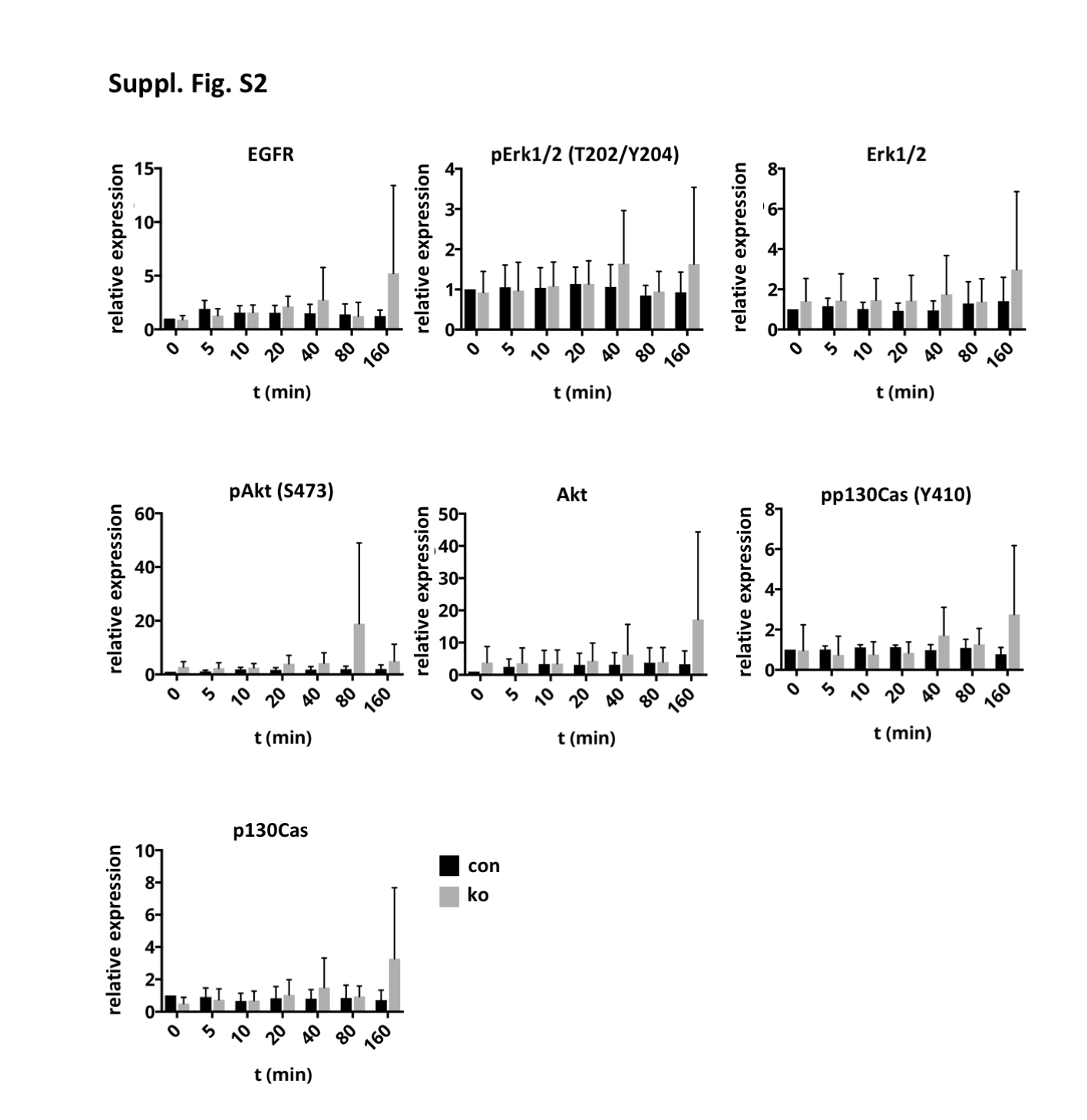

Supplement: Supplementary file 2 — Fig. S2. Normal EGFR signaling in the breast cancer cell line MDA‐MB‐231 lacking Ack. Quantification of Western blots for indicated proteins of lysates of MDA‐MB‐231 breast cancer cells with (con) or without (ko) ACK1, stimulated with EGF for indicated times, for, with corresponding quantifications (n: 3/3). [file FEB4-11-1579-s001.tiff]

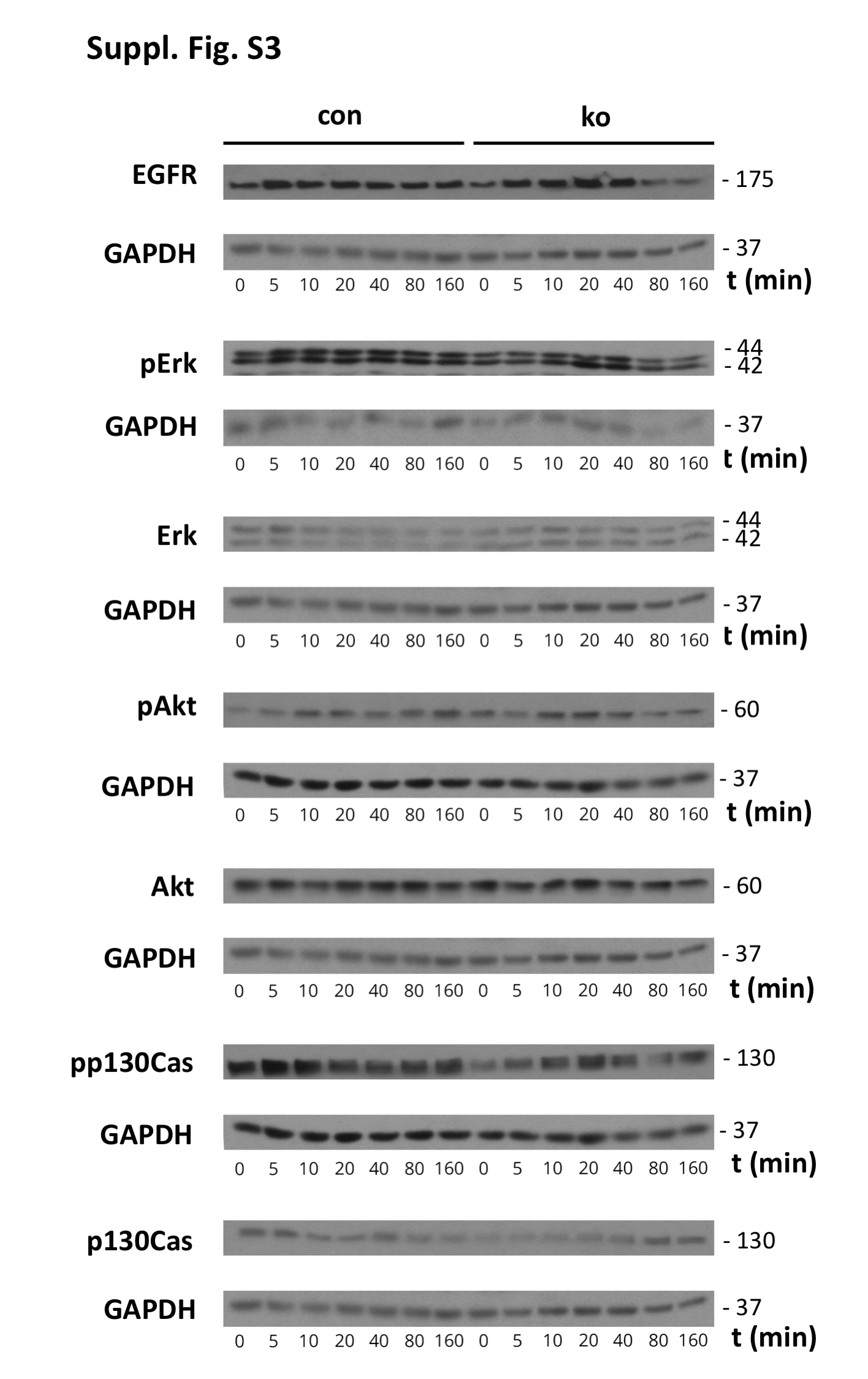

Supplement: Supplementary file 3 — Fig. S3. Representative Western blots for Fig. S2 [file FEB4-11-1579-s002.tiff]
